# Supplementary material for: Enhancing Prediction Performance by Add-On Combining Circulating Tumor Cell Count, CD45neg EpCAMneg Cell Count on Colorectal Cancer, Advance, and Metastasis
Source: Cancers (Basel). 2021 May 21;13(11):2521. doi: 10.3390/cancers13112521 (PMC8196640; doi:10.3390/cancers13112521)
Supplement: Supplementary file 1 [file cancers-13-02521-s001.zip › cancers-1169819-supplementary.pdf]

cancers MDPI

Supplementary Material: Enhancing Prediction Performance by Add-on Combining Circulating Tumor Cell Count, CD45^{neg} EpCAM^{neg} Cell Count on Colorectal Cancer, Advance, and Metastasis

Sherry Yueh-Hsia Chiu, Chia-Hsun Hsieh, Jeng-Fu You, Po-Yu Chu, Hsin-Yuan Hung, Pao-Hsien Chu and Min-Hsien Wu

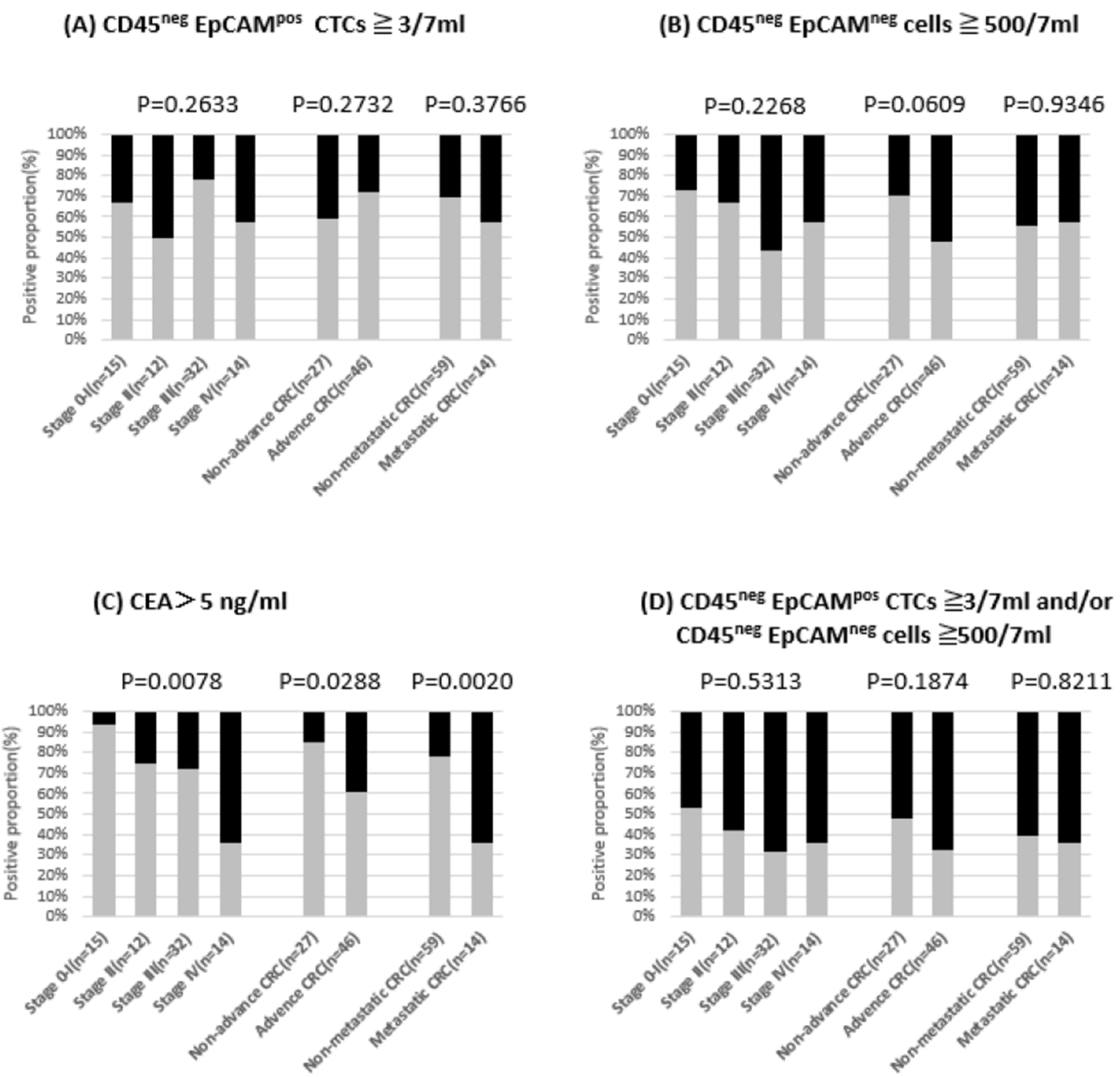

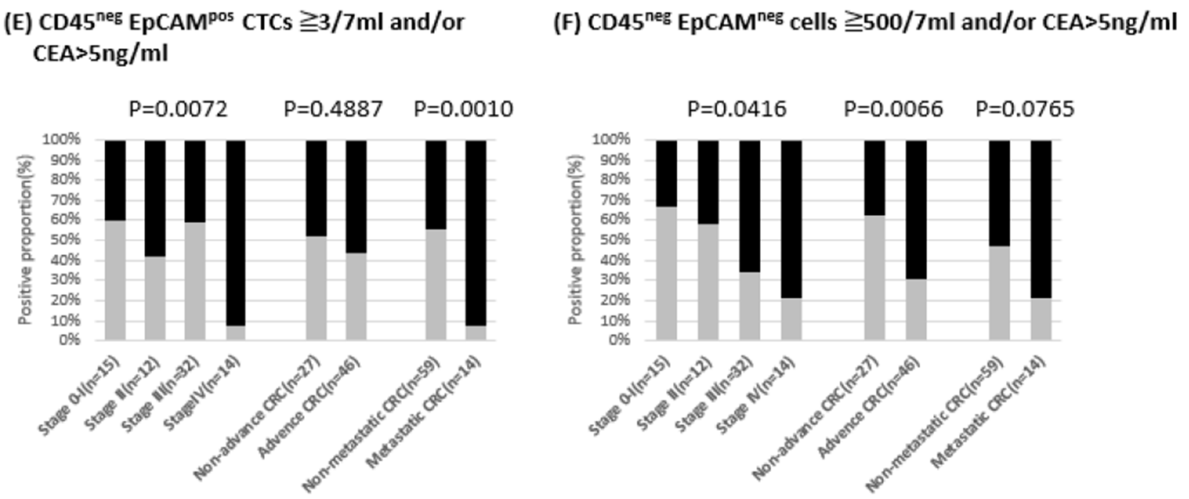

Figure S1. The proportional distribution comparison by advanced or metastasis statuses using single or combing different biomarkers.

Table S1. Performance of CEA, CD45neg EpCAMpos CTCs (continuous), CD45neg EpCAMneg (≧400) cell counts, and combined biomarkers predicting advanced or metastasis CRCs.

Biomarker(s)	Predictor(s)*			Advanced vs. Non-advanced CRC AUROC (95%CI)	Metastasis vs. Non-metastasis CRC AUROC (95%CI)
	$CD45^{neg}EpCAM^{pos}$ CTCs (continuous)	$CD45^{neg}$ $EpCAM^{neg}$ cells ≧400/7ml	CEA>5ng/ml		
1-type	√			0.561 (0.424, 0.699)	0.664 (0.497, 0.831)
		√		0.635 (0.491, 0.779)	0.630 (0.475, 0.784)
			√	0.643 (0.515, 0.771)	0.780 (0.645, 0.914)
2-type	√	√		0.638 (0.494, 0.781)	0.662 (0.495, 0.830)
		√	√	0.712 (0.587, 0.836)	0.786 (0.661, 0.911)
	√		√	0.635 (0.506, 0.763)	0.837 (0.740, 0.934)
3-type	√	√	√	0.710 (0.586, 0.835)	0.837 (0.739, 0.935)

* all analysis was adjusted age (continuous) and gender. AUROC: area under the receiver operating characteristic. CEA: Carcinoembryonic Antigen.

Table S2. Performance of CEA, CD45neg EpCAMpos CTCs (continuous), CD45neg EpCAMneg (≧500) cell counts, and combined biomarkers predicting advanced or metastasis CRCs.

Biomarker(s)	Predictor(s)*			Advanced vs. Non-advanced CRC AUROC (95%CI)	Meta vs. Non-meta CRC AUROC (95%CI)
	$CD45^{neg}$ $EpCAM^{pos}$ CTCs (continuous)	$CD45^{neg}$ $EpCAM^{neg}$ cells ≧500/7ml	CEA>5ng/ml		
1-type	√			0.561(0.424, 0.699)	0.664(0.497, 0.831)
		√		0.658(0.518, 0.798)	0.627(0.478, 0.777)
			√	0.643(0.515, 0.771)	0.780(0.645, 0.914)
2-type	√	√		0.658(0.519, 0.797)	0.668(0.504, 0.833)
		√	√	0.709(0.584, 0.834)	0.782(0.650, 0.914)
	√		√	0.635(0.506, 0.763)	0.837(0.740, 0.934)
3-type	√	√	√	0.710(0.585, 0.835)	0.826(0.706, 0.945)

* all analysis was adjusted age (continuous) and gender. AUROC: area under the receiver operating characteristic. CTC: Circulating Tumor Cells.

Table S3. Performance of CEA, CD45neg EpCAMpos (≧3) CTCs, CD45neg EpCAMneg (≧400) cell counts, and combined biomarkers predicting advanced or metastasis CRCs.

Biomarker(s)	Predictor(s)*			Advanced vs. Non-advanced CRC AUROC (95%CI)	Meta vs. Non-meta CRC AUROC (95%CI)
	$CD45^{neg}$ $EpCAM^{pos}$ CTCs ≧3/7ml	$CD45^{neg}$ $EpCAM^{neg}$ cells ≧400/7ml	CEA>5ng/ml		
1-type	√			0.614(0.478, 0.749)	0.642(0.487, 0.797)
		√		0.635(0.491, 0.779)	0.630(0.475, 0.784)
			√	0.643(0.515, 0.771)	0.780(0.645, 0.914)
2-type	√	√		0.672(0.542, 0.803)	0.639(0.473, 0.805)
		√	√	0.712(0.587, 0.836)	0.786(0.661, 0.911)
	√		√	0.677(0.554, 0.800)	0.815(0.710, 0.920)
3-type	√	√	√	0.727(0.609, 0.845)	0.818(0.714, 0.922)

* all analysis was adjusted age (continuous) and gender. AUROC: area under the receiver operating characteristic. CTC: Circulating Tumor Cells.

Table S4. Performance of CEA, CD45neg EpCAMpos (≧3) CTCs, CD45neg EpCAMneg (≧500) cell counts, and combined biomarkers predicting advanced or metastasis CRCs.

Biomarker(s)	Predictor(s)*			Advanced vs. Non-advanced CRC	Meta vs. Non-meta CRC
	CD45^{neg} EpCAM^{pos} CTCs ≧3/7ml	CD45^{neg} EpCAM^{neg} cells ≧500/7ml	CEA>5ng/ml	AUROC (95%CI)	AUROC (95%CI)
1-type	√			0.614(0.478, 0.749)	0.642(0.487, 0.797)
		√		0.658(0.518, 0.798)	0.627(0.478, 0.777)
			√	0.643(0.515, 0.771)	0.780(0.645, 0.914)
2-type	√	√		0.680(0.553, 0.807)	0.639(0.484, 0.794)
		√	√	0.709(0.584, 0.834)	0.782(0.650, 0.914)
	√		√	0.677(0.554, 0.800)	0.815(0.710, 0.920)
3-type	√	√	√	0.713(0.594, 0.833)	0.812(0.700, 0.925)

* all analysis was adjusted age (continuous) and gender. AUROC: area under the receiver operating characteristic. CTC: Circulating Tumor Cells.

Table S5. Performance of CEA, CD45neg EpCAMpos (≧3) CTCs, CD45neg EpCAMneg (≧600) cell counts, and combined biomarkers predicting advanced or metastasis CRCs.

Biomarker(s)	Predictor(s)*			Advanced vs. Non-advanced CRC	Meta vs. Non-meta CRC
	CD45^{neg} EpCAM^{pos} CTCs ≧3/7ml	CD45^{neg} EpCAM^{neg} cells ≧600/7ml	CEA>5ng/ml	AUROC (95%CI)	AUROC (95%CI)
1-type	√			0.614(0.478, 0.749)	0.642(0.487, 0.797)
		√		0.666(0.529, 0.803)	0.617(0.459, 0.776)
			√	0.643(0.515, 0.771)	0.780(0.645, 0.914)
2-type	√	√		0.681(0.557, 0.805)	0.628(0.467, 0.790)
		√	√	0.709(0.584, 0.833)	0.785(0.652, 0.917)
	√		√	0.677(0.554, 0.800)	0.815(0.710, 0.920)
3-type	√	√	√	0.717(0.598, 0.836)	0.816(0.705, 0.928)

* all analysis was adjusted age (continuous) and gender. AUROC: area under the receiver operating characteristic. CTC: Circulating Tumor Cells.
